# Plasma metabolomics signatures of developmental dysplasia of the hip in Tibet plateau

**DOI:** 10.1186/s13023-024-03230-w

**Published:** 2024-06-08

**Authors:** Ye Guo, Xiaogang Li, De Yang, Nyima Yedron, Tao Chen, Jianchu Li, Yanming Lei, Ping Li, Jiamei Ji, Li Shi, Xiao Yang, Ten Cho

**Affiliations:** 1 Department of Clinical Laboratory, State Key Laboratory of Complex Severe and Rare Diseases, Peking Union Medical College Hospital, Chinese Academy of Medical Science and Peking Union Medical College, Beijing, China; 2 Medical Science Research Center, State Key Laboratory of Complex Severe and Rare Diseases, Peking Union Medical College Hospital, Chinese Academy of Medical Science and Peking Union Medical College, Beijing, China; 3 Department of Ultrasound, People’s Hospital of Tibet Autonomous Region, No. 16, North Linkuo Road, Lhasa, Tibet 850000 China; 4 Department of Ultrasound, Beijing Jishuitan Hospital, The 4th Clinical College, Peking University, Xinjiekou Dongjie, Xicheng District, Beijing, 100035 China; 5 Department of Ultrasound, State Key Laboratory of Complex Severe and Rare Diseases, Peking Union Medical College Hospital, Chinese Academy of Medical Science and Peking Union Medical College, No. 1 Shuaifuyuan, Dong Cheng District, Beijing, 100730 China; 6 Department of Radiology, People’s Hospital of Tibet Autonomous Region, No. 16, North Linkuo Road, Lhasa, Tibet 850000 China; 7 Department of Orthopedics, People’s Hospital of Tibet Autonomous Region, No. 16, North Linkuo Road, Lhasa, Tibet 850000 China; 8 Department of Infectious Diseases, People’s Hospital of Tibet Autonomous Region, No.16, North Linkuo Road, Lhasa, Tibet 850000 China; 9 Department of Laboratory Medicine, People’s Hospital of Tibet Autonomous Region, No.16, North Linkuo Road, Lhasa, Tibet 850000 China

**Keywords:** Developmental dysplasia of the hip, Metabolomics, Biomarker, Rare disease

## Abstract

**Background:**

Developmental dysplasia of the hip (DDH) is a common childhood health complaint, whose etiology is multifactorial. The incidence of DDH is variable and higher in Tibet plateau. Here, we collected plasma samples and studied the metabolomics signatures of DDH.

**Methods:**

Fifty babies were enrolled: 25 with DDH and 25 age-matched non-DDH healthy controls (HC group). We collected plasma samples, laboratory parameters and conducted untargeted metabolomics profiling.

**Results:**

There are many differential metabolites among patients with DDH, including 4-β-hydroxymethyl-4-α-methyl-5-α-cholest-7-en-3-beta-ol, β-cryptoxanthin, α-tocopherol, taurocholic acid, glycocholic acid, 2-(3,4-dihydroxybenzoyloxy)-4,6-dihydroxybenzoate, arabinosylhypoxanthine, leucyl-hydroxyproline, hypoxanthine. The main differential metabolic pathways focused on primary bile acid biosynthesis, arginine and proline metabolism, phenylalanine metabolism, histidine metabolism, purine metabolism.

**Conclusions:**

To our knowledge, this is the first report of metabolomics profile in babies with DHH. By combining the α-tocopherol and taurocholic acid, we could achieve the differential diagnosis of DDH.

**Supplementary Information:**

The online version contains supplementary material available at 10.1186/s13023-024-03230-w.

## Introduction

Developmental dysplasia of the hip (DDH) is a common childhood hip joint health complaint, including acetabular dysplasia, hip dislocation, and subluxation. A previous epidemiological investigation revealed a marked elevation in the incidence of DDH in the Tibetan region as compared to lowland areas, suggesting that altitude may play a significant role in the risk factors associated with DDH [1]. The pathogenesis of DDH is complex and involves multiple factors, including genetic and environmental factors. The most common risk factors for DDH include female gender, first birth, multiple births, family history of DDH, breech presentation, oligohydramnios, feet inward rotation deformity. The clinical manifestations of DDH vary with the age and severity of the dislocation. Children with unilateral dislocation may have limb length inequality, while children with bilateral hip dislocation may have a limping gait or duck-like gait. Additionally, children with DDH often have asymmetric thigh wrinkle.

The treatment for developmental dysplasia of the hip (DDH) varies depending on the age and severity of the condition. Generally, the goal is to achieve concentric reduction of the hip joint to provide a favorable environment for the development of the femoral head and acetabulum. For newborns and infants under 6 months of age, the most commonly used treatment method is the use of Pavlik’s splint. For Ortolani-positive hips, the recovery rate can reach 95%. After 6 months, the failure rate may exceed 50%. For the first 3 weeks of treatment, the splint should be reexamined and the ultrasound should be performed weekly. If the hip joint is reduced and stable, the follow-up interval can be extended until the ultrasound examination returns to normal.For patients between 6 months and 18 months of age, hip subluxation or dislocation should be treated by closed or open reduction as the preferred treatment method. For acetabular dysplasia, bracing can be used. For patients older than 18 months, surgical treatment is often required. The surgical approach will be individualized based on the patient’s age to achieve the best treatment outcome. For patients between 18 months and 8 years of age, open reduction and acetabular osteotomy are recommended. It is important to note that if DDH is not treated promptly, it can lead to serious complications and sequelae such as deformity, limited function, and pain. Therefore, early diagnosis and treatment are recommended to achieve a good prognosis. The early treatment of DDH is important to obtain a normal or near-normal hip [2, 3].

Finding abnormalities through early screening and timely correction by physical methods can effectively avoid the need for long-term hip replacement and reduce the long-term disability rate [4, 5]. The Standing Medical Advisory Committee (SMAC) was established by legislation in 1949 as source of relatively independent clinical advice for government of United Kingdom. The SMAC recommends that clinical screening for DDH should be conducted for all newborns, which emphasizes multiple examinations including on the day of birth, at discharge, 6 weeks, 6–9 months, and after starting to walk [6–9]. Magnetic resonance imaging (MRI) can be used to diagnose DDH in children with limited hip abduction, limb length inequality, limping, or duck-like gait. Ultrasound screening is an important method for early diagnosis, which can evaluate the shape of the hip joint, the position of the femoral head and the stability of the hip joint. Graf examination is the earliest ultrasound examination method to measure the coronal section of the hip joint, and it is also currently recognized as the most effective screening and diagnosis method. This method measures the α angle and β angle, which represent the acetabular angle and cartilage part angle, respectively. As age increases, the acetabular index decreases, and by 2 years of age it should be within 24° [10–14].

However, ultrasound screening requires high professional and technical capabilities, and there is a proportion of misdiagnosis and missed diagnosis [8, 9]. The early diagnosis is difficult due to the insidious clinical features and mild early symptoms of DDH, therefore, biomarkers are urgently needed for auxiliary diagnosis.

Metabolomics can achieve in-depth investigation of metabolites, and has been used to develop biomarkers for early disease diagnosis and monitoring therapy. An explorative study into the aetiology of DDH using targeted urine metabolomics showed that DDH is a systemic disease associated with altered uptake, formation, or handling of sulphate [15, 16]. There are no reports on metabolomics research using blood samples of DDH. Here, we performed integrated metabolomics profiling of patients with DDH.

## Materials and methods

### Participants

The study involved a cohort of 25 Tibetan pediatric patients diagnosed with DDH at the People’s Hospital of Tibet between January 2021 and March 2022. Inclusion in the study required a confirmed diagnosis of one of the following: (1) a complete hip joint dislocation, with no contact between the original articulating surfaces; (2) a hip joint subluxation, with partial contact maintained between the articulating surfaces; or (3) acetabular dysplasia, characterized by insufficient development of the acetabulum. The diagnosis was rendered by seasoned pediatric orthopedic specialists based on comprehensive medical evaluations, which included assessments of abnormal gait patterns such as limping or Trendelenburg gait. Physical examinations for asymmetrical gluteal folds or thigh skin creases were also conducted. Additional clinical signs considered were the pronounced elevation and lateral displacement of the greater trochanters, restricted abduction of the hip joint, and positive Ortolani, Barlow, Allis, or Galeazzi signs. Diagnostic confirmation was achieved through X-ray imaging. Cases with concurrent chronic diseases or other musculoskeletal disorders were meticulously excluded to maintain the study’s focus on isolated DDH. The control group comprised healthy Tibetan children who underwent routine health screenings at the People’s Hospital of Tibet concurrently with the study period and exhibited no abnormalities. The selection of control subjects was age and gender-matched to the case group to ensure comparability.

Fasting blood samples were collected. Centrifuge the blood at 3000 rpm for 10 min at 4 °C, then remove the supernatant and sub-package it into 1.5 mL centrifuge tubes. Finally, freeze the sample at -80 °C. The clinical data was described in Table 1.

### Instrument and materials

Mass spectrometry grade methanol, ammonium acetate, acetonitrile, and ammonium hydroxide were from Sigma Aldrich. ACQUITY UPLC BEH Amide column (1.7 μm, 2.1 mm× 100 mm, Waters Corporation, Milford, MA, USA) was used for separation. Vanquish liquid chromatography system coupled with Orbitrap Exploris 120 (Thermo Fisher Scientific) was used for metabolomics profiling.

### Specimen preparation

Fifty microliters plasm was blended with 200 µL methanol/acetonitrile solution (1:1, v/v) containing isotopically-labelled internal standard mixture in a sterile EP tube. The samples were vortexed and sonicated for 10 min in ice-water bath. After incubation at -40 °C for 1 h, the samples were centrifuged at 13,800 g for at 4 °C 15 min. The supernatant was used for mass spectrometry detection. Equal volume of all samples was mixed to make quality control (QC) sample.

### LC-MS/MS analysis

Vanquish liquid chromatography system coupled with Orbitrap Exploris 120 (Thermo Fisher Scientific) was used for metabolomics profiling on information-dependent acquisition (IDA) mode. UPLC BEH Amide column (1.7 μm, 2.1 mm× 100 mm, Waters Corporation, Milford, MA, USA) was used for separation. The mobile phase A was 25 mmol/L ammonium acetate and 25 ammonia hydroxide in water(pH = 9.75), mobile phase B was acetonitrile. The autosampler temperature was 4 °C, and the injection volume was 2 µL. The ESI source conditions were: Aux gas flow rate, 15 Arb; sheath gas flow rate, 50 Arb; full MS resolution,60,000; capillary temperature, 320 ℃.

### Data analysis

For metabolomics analysis, the mzXML format transition of raw MS data was used with ProteoWizard. Peak picking, extraction, integration, and alignment were performed using R and XCMS software. Identification of metabolites was performed by comparing in-house MS2 database (Biotree DB). First, we carried out a series of data management, including: (1) off-value filtering. (2) Missing value filtering. (3) Missing values fill. (4) Data normalization. The internal standard (IS) is used for normalization. The statistically significant criterion was: variable importance in the projection (VIP) value > 1, p values ≤ 0.05. MetaboAnalyst 5.0 (Xia Lab @ McGill Sweden) was used to conduct heat map, pathway and biomarker analysis.

## Results

### Clinical characteristics of patients with DDH

The average age of the children enrolled was 19.9 ± 9.1 months. The altitude of residence was 4101.9 ± 344.7 m. None had a family history of DDH. The ratio of girls to boys was 18:7. There were 2 cases (8%) of oligohydramnios during the fetal period, 1 case (4%) of post-term birth, and 23 cases (92%) were vaginal deliveries, all of which were cephalic presentations. Four (16%) were the first birth of the mother. The average birth weight was 3.5 ± 0.5 kg, with 9 (36%) weighing more than 4 kg at birth. Eleven (44%) were swaddled with tight wrappings, such as candle wraps during infancy. Among the 25 children, the number of cases with affected left hip, right hip, or both hips were 7, 8, and 11, respectively. Twenty-two (88%) underwent open surgical treatment after diagnosis. (Table 1).

**Table 1:** Clinical characteristics of patients with developmental dysplasia of the hip (DDH)

	*N* = 25
Age(months)	19.9 ± 9.1
Mean Altitude of Residence (m)	4101.9 ± 344.7
Family History of DDH (%)	0 (0%)
Gender (Female/Male)	(18/7)
Oligohydramnios (%)	2 (8%)
Post-term Birth (%)	1 (4%)
Vaginal Delivery (%)	23 (92%)
First Delivery (%)	4 (16%)
Breech Delivery (%)	0 (0%)
Birth Weight (kg)	3.5 ± 0.5 kg
Neonatal Macrosomia (%)	9 (36%)
Tight Swaddled or Twined Baby Wraps (%)	11 (44%)
Affected Hip Joint(s) (Left /Right/Bilateral)	6/6/13
Underwent Open Surgical Treatment (%)	22 (88%)

### OPLS-DA and volcano map

**Fig. 1:**
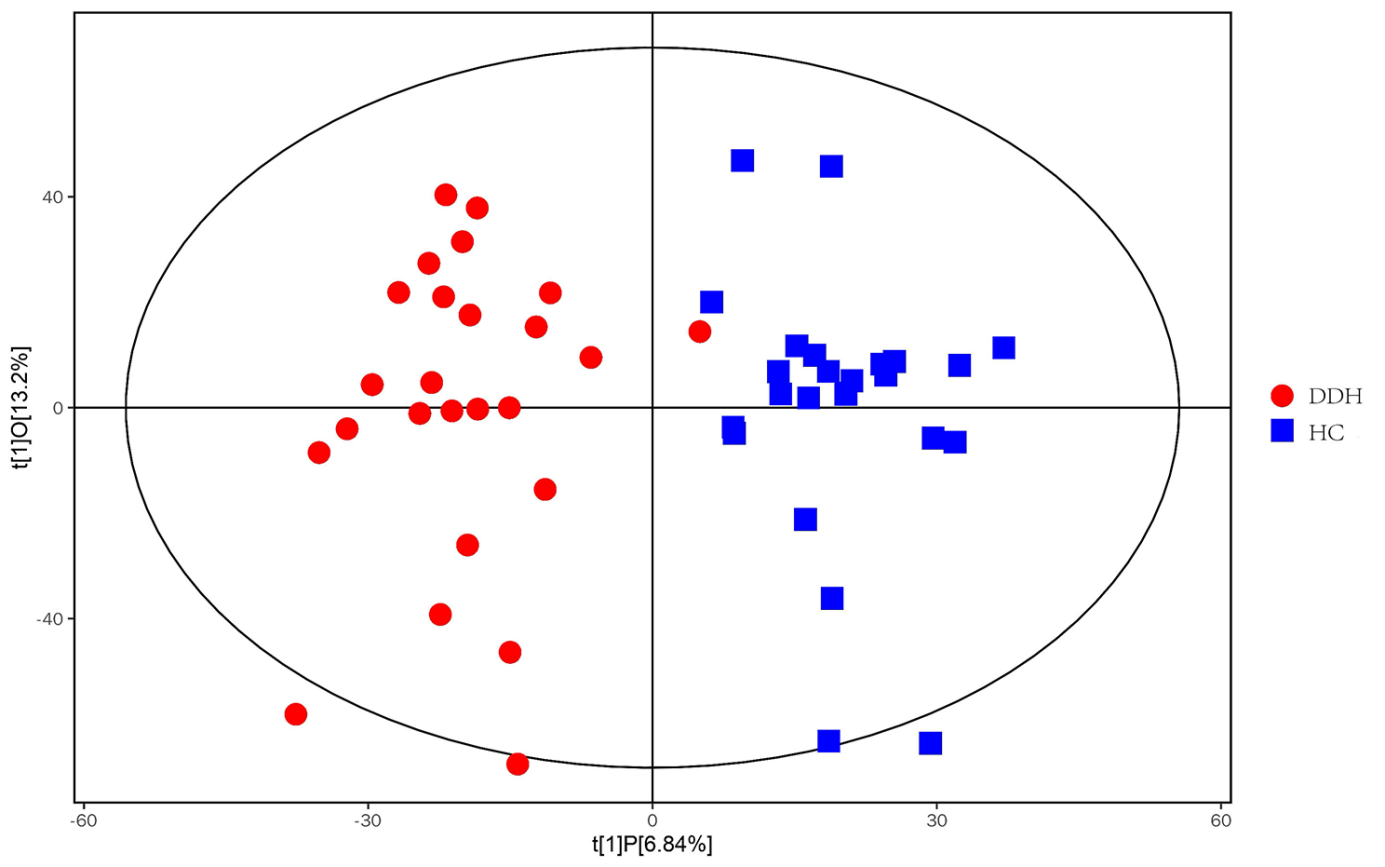
OPLS-DA model of metabolomics in ESI+/ESI– ionization mode. ESI+: electrospray ionization at positive mode; ESI–: electrospray ionization at negative mode

**Fig. 2:**
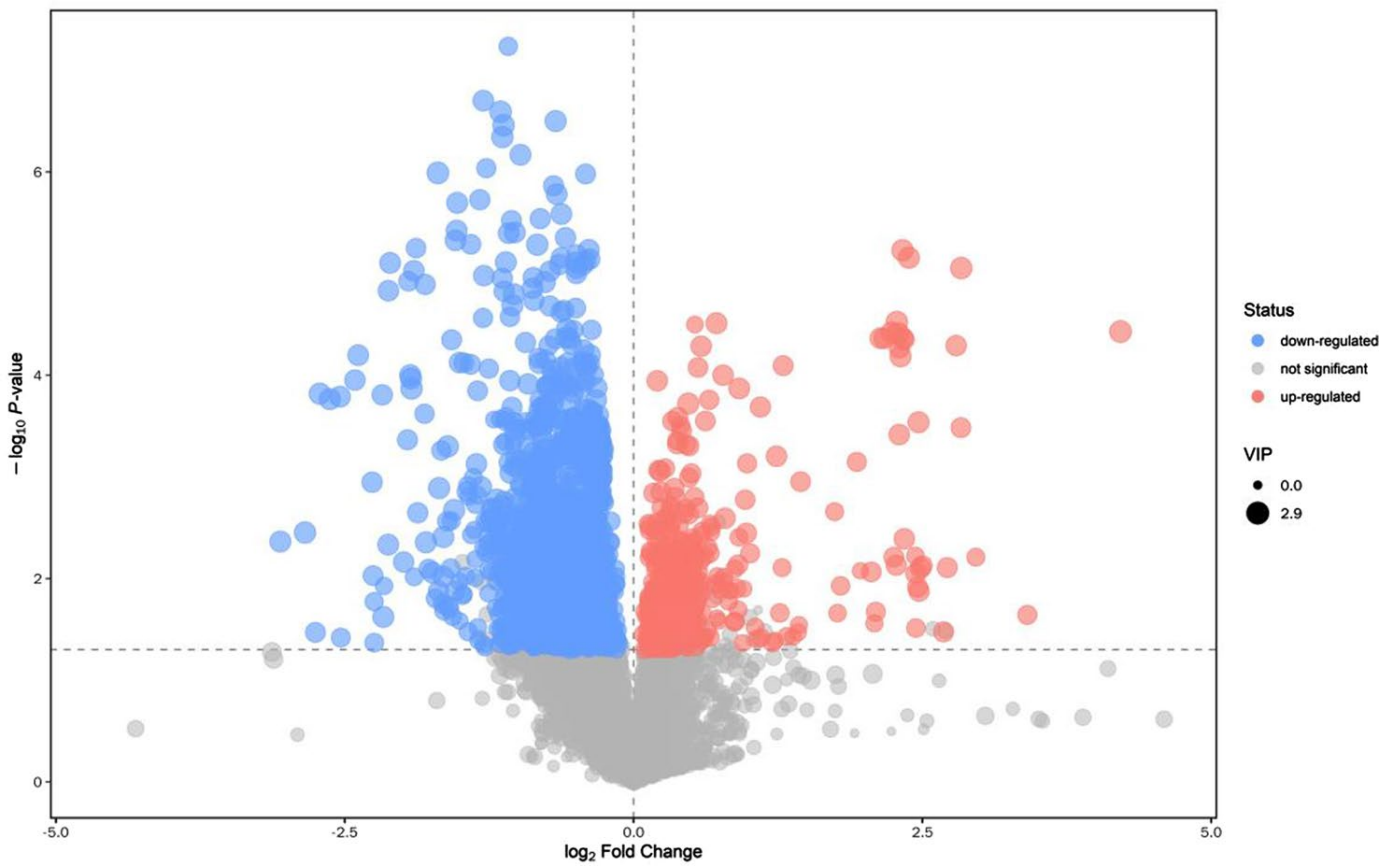
Volcano plot of metabolomics in ESI + ionization mode

Through orthogonal projections to latent structures- discriminant analysis (OPLS-DA), we can get more reliable information about the difference between groups. In the figure, horizontal coordinate t [1]P represents the differences between sample groups; vertical coordinate t [1]O represents differences within sample groups. As can be seen from the score chart, the difference is significant, and most samples are in 95% confidence interval (Fig. 1). The volcano map show distribution of metabolites. The card value criteria are based on the following two indicators: P-value < 0.05; VIP>1. Each point represents a peak in the volcano map (Fig. 2).

### Heatmap and matchstick diagram

**Fig. 3:**
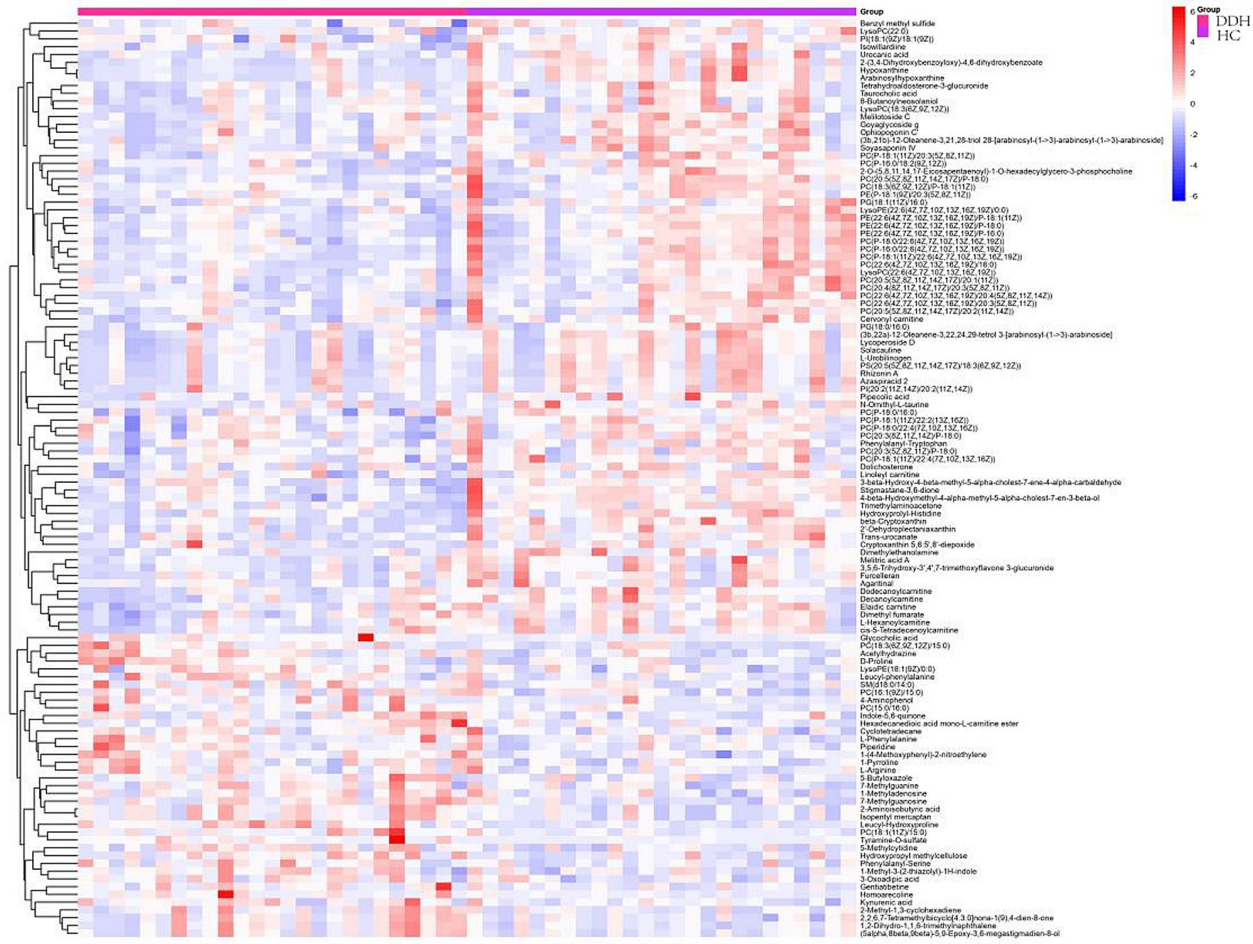
Heatmap of the top different features in metabolomics

**Fig. 4:**
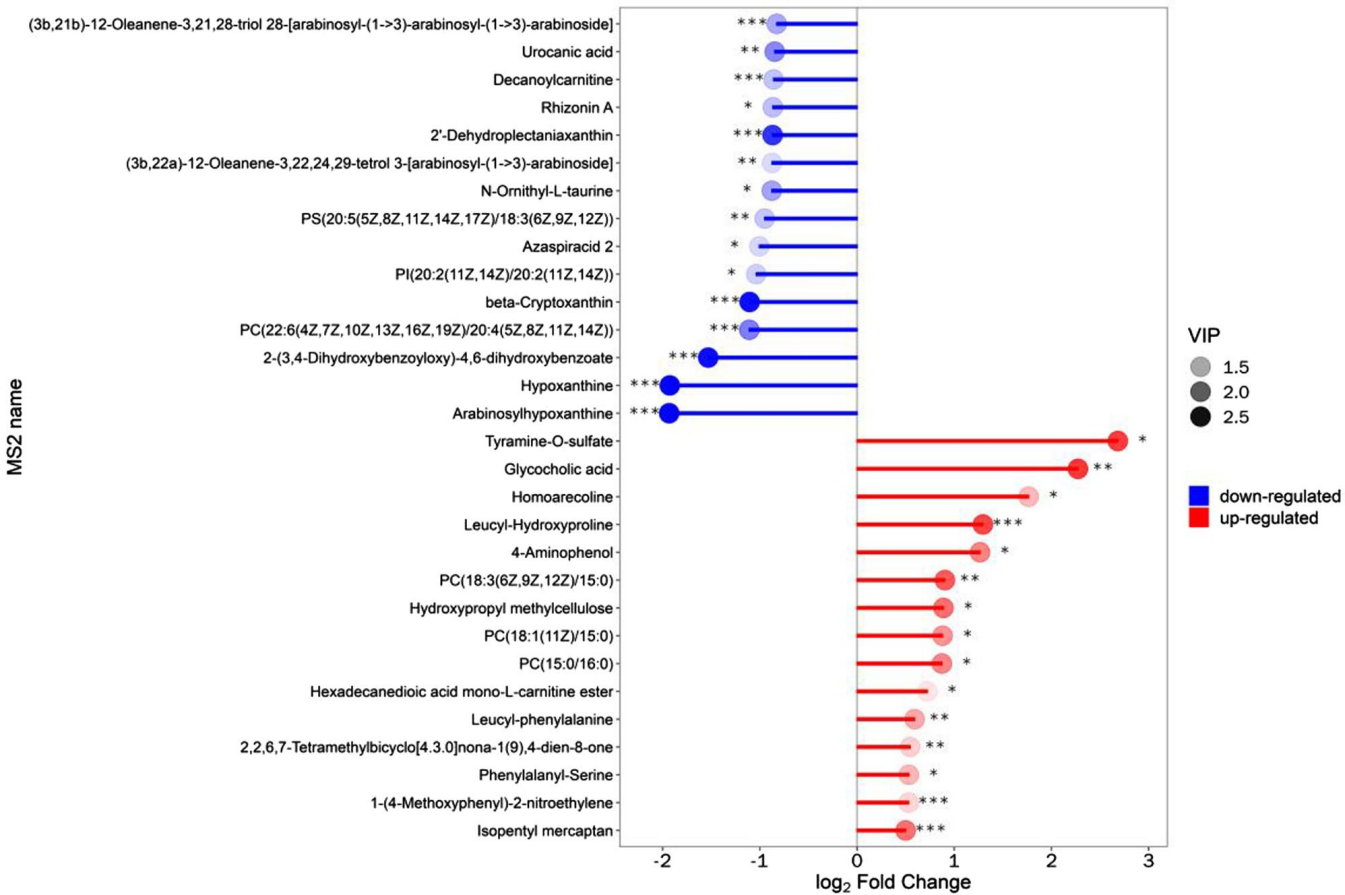
Matchstick diagram for the DDH vs. HC groups

**Fig. 5:**
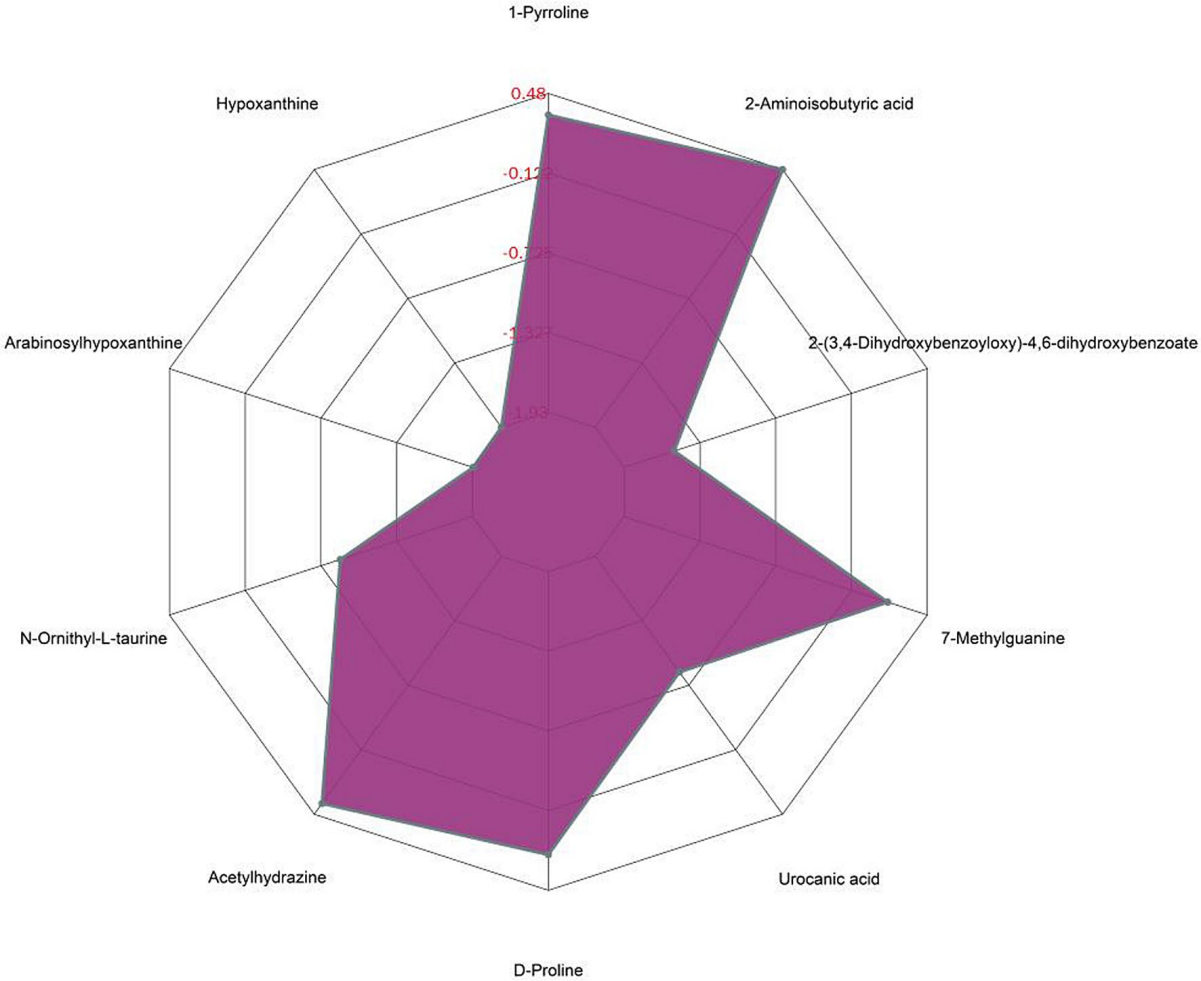
Radar chart analysis for the DDH vs. HC groups

We calculate the quantitative values and cluster the differential metabolites, using heat maps to display (Fig. 3). β-cryptoxanthin, α-tocopherol, taurocholic acid, glycocholic acid, 2-(3,4-Dihydroxybenzoyloxy)-4,6-dihydroxybenzoate, arabinosylhypoxanthine, leucyl-hydroxyproline, hypoxanthine, stigmastane-3,6-dione were the main differential metabolites. The top 15 multiples of change up-modulation and down-modulation are selected to display the results using matchstick diagram. The analysis showed the different metabolites with a large degree of change, so the gene expression of the corresponding enzyme could be strongly activated or strongly inhibited (* 0.01 < *p* < 0.05, ** 0.001 < *p* < 0.01, *** *p* < 0.001, *** *P* < 0.001), and verification could be carried out for the regulation of enzyme gene expression by some metabolites related to the subject (Fig. 4).The radar chart is present with red fonts, grid line shows difference multiple, and purple shadows are composed of the difference multiple lines for each substance (Fig. 5).

### KEGG enrichment and pathway analysis

**Fig. 6:**
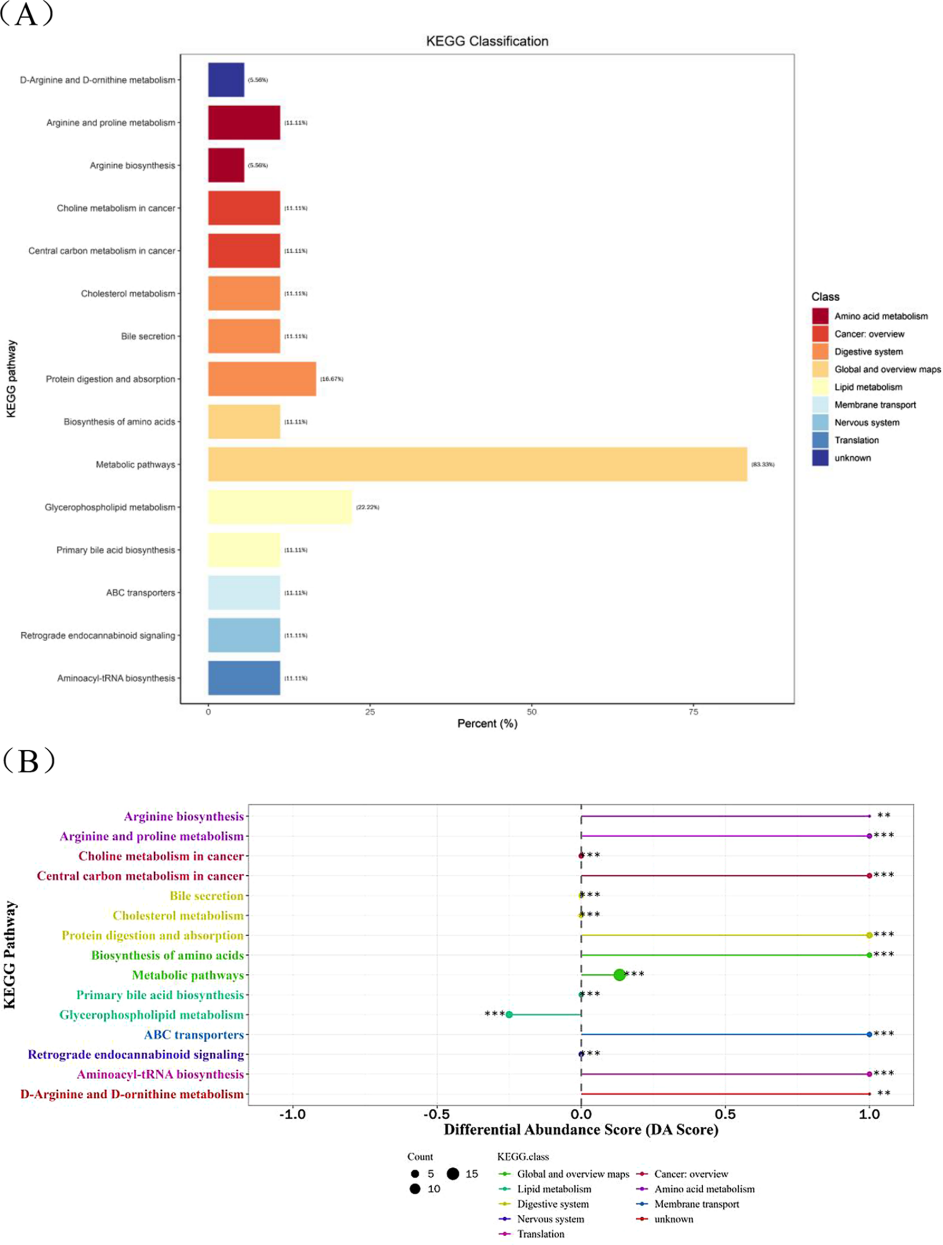
(**A**) KEGG enrichment analysis for the DDH vs. HC groups. (**B**) Differential abundance score analysis for the DDH vs. HC groups

Kyoto Encyclopedia of Genes and Genomes (KEGG) Pathway database was used for pathway network analysis. We collated all pathways corresponding to the differential metabolites mapping of the species Homo sapiens (human), and the KEGG pathway annotation information table contains the following information: the name of the metabolic pathway enriched with metabolites; the number of differential metabolites in the pathway; and the number of all metabolites detected in the pathway. After obtaining the above results, we marked the differential metabolites on the KEGG pathway map (Fig. 6A). Differential abundance score (DA Score) represents the overall change degree of metabolites (Fig. 6B). The global metabolic pathways are up regulated, including arginine biosynthesis, protein digestion and absorption, arginine and proline metabolism, ABC transporters, central carbon metabolism in cancer, biosynthesis of amino acids, D-arginine and D-ornithine metabolism, aminoacyl-tRNA biosynthesis. Only glycerophospholipid metabolism is down regulated.

**Fig. 7:**
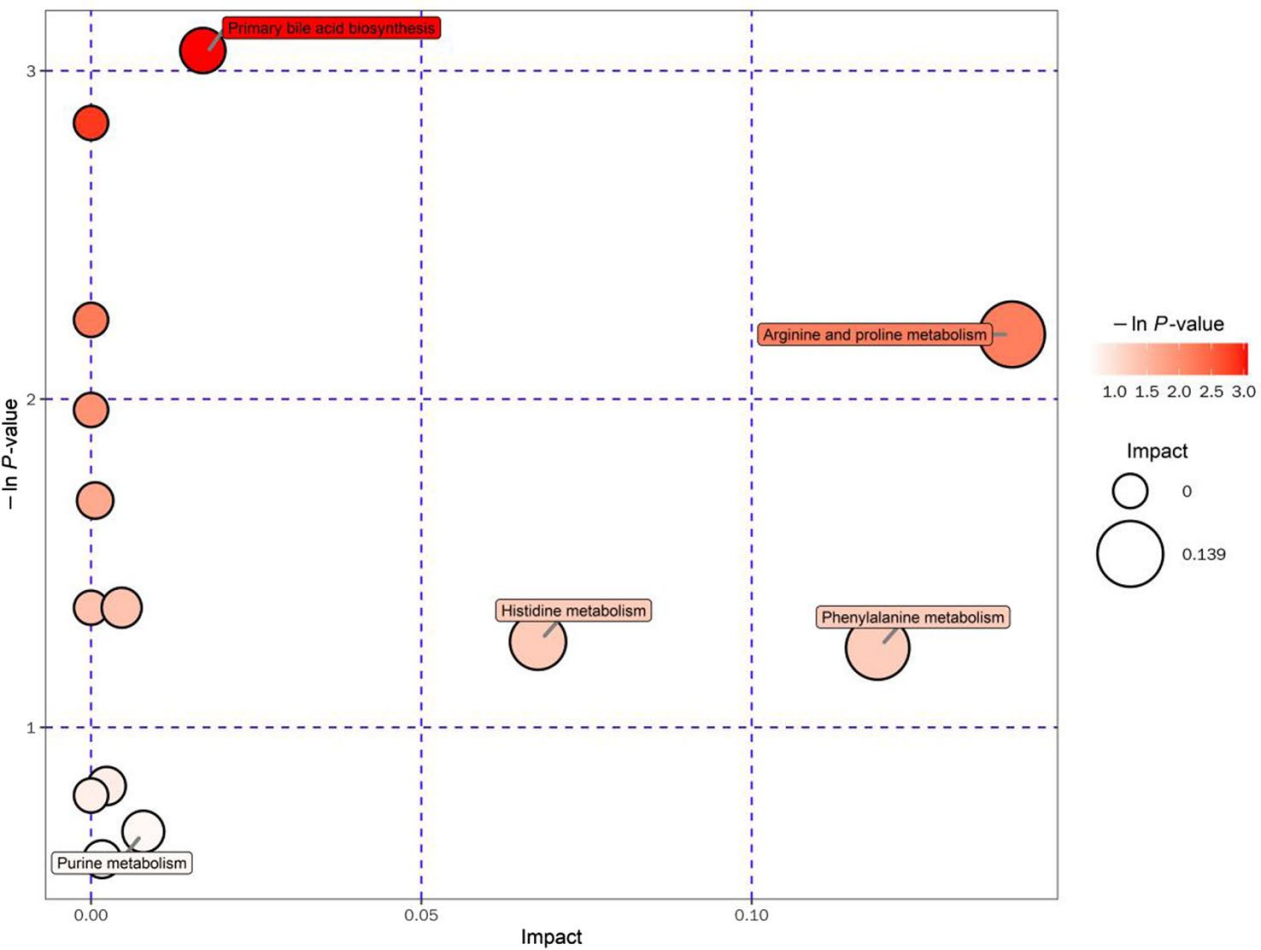
Bubble plot for group the DDH vs. HC groups

**Fig. 8:**
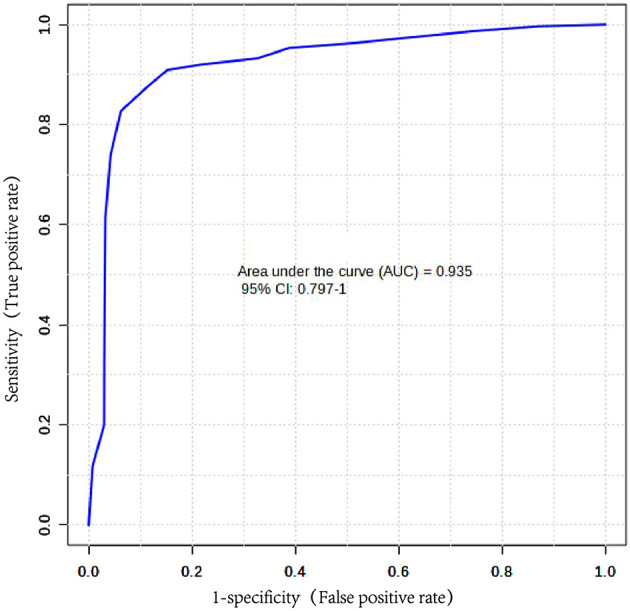
ROC curve of α-tocopherol and taurocholic acid

In the bubble diagram, every bubble shows a metabolic pathway, and the size represent the influence factor. The main differential metabolic pathways focused on primary bile acid biosynthesis, phenylalanine metabolism, histidine metabolism and arginine and proline metabolism (Fig. 7). In bioinformatics, area under the curve (AUC) is commonly used to evaluate the performance of classification models. It is a value between 0 and 1, which measures the model’s ability to classify samples. Generally, the closer the AUC is to 1, the higher the authenticity of the detection method; when the AUC is equal to 0.5, the authenticity is the lowest and has no application value. Here, we evaluated the AUC of different metabolites (Table 2). By combining the α-tocopherol and taurocholic acid, we could achieve the differential diagnosis of DDH (AUC = 0.935)(Figure 8).

**Table 2:** AUC of different metabolites

MS name	AUC
4-β-Hydroxymethyl-4-α-methyl-5-α-cholest-7-en-3-beta-ol	0.8784
β-Cryptoxanthin	0.8784
α-Tocopherol	0.8736
Taurocholic acid	0.8672
Glycocholic acid	0.8656
2-(3,4-Dihydroxybenzoyloxy)-4,6-dihydroxybenzoate	0.864
Arabinosylhypoxanthine	0.8624
Leucyl-hydroxyproline	0.8592
Hypoxanthine	0.8592
Stigmastane-3,6-dione	0.8544
Tyramine-O-sulfate	0.8496

## Discussion

Developmental dysplasia of the hip (DDH) is a common childhood hip joint health complaint, encompassing acetabular dysplasia, hip dislocation, and subluxation. Timely treatment is crucial to achieving a normal or near-normal hip. Graf examination, the earliest ultrasound method for assessing the hip joint’s coronal section, is currently the most effective screening and diagnostic tool. However, ultrasound screening demands advanced professional and technical expertise, leading to a proportion of misdiagnoses and missed diagnoses [17–21]. The subtle clinical features and mild early symptoms of DDH pose challenges for early diagnosis. Therefore, we employed metabolomics to identify DDH biomarkers.

In this project, we conducted untargeted metabolomics to discover potential biomarkers of DDH. β-Cryptoxanthin, α-tocopherol, taurocholic acid, glycocholic acid, 2-(3,4-Dihydroxybenzoyloxy)-4,6-dihydroxybenzoate, arabinosylhypoxanthine, leucyl-hydroxyproline, hypoxanthine, and stigmastane-3,6-dione emerged as the principal differential metabolites. (Fig. 7).

4-β-Hydroxymethyl-4-α-methyl-5-α-cholest-7-en-3-beta-ol is part of the steroid biosynthesis pathway. Glycocholic acid is secondary bile acid, palys a role in the absorption of fat as detergent. 2-(3,4-dihydroxybenzoyloxy)-4,6-dihydroxybenzoate belongs to the flavonoid metabolism. Taurocholic acid plays a role in regulation of critical enzymes associated with cholesterol homeostasis. It is used as a cholagogue and choleretic. Ping Li et al. found that taurocholic acid sodium hydrate could induce chondrogenesis and osteogenesis in mesenchymal stem cells (MSCs), ameliorating the secondary osteoporosis and delay the progression of intervertebral disc degeneration (IDD) in mice by targeting MAPK3 [22]. In our research, we also found that the patients with DDH had lower concentration of taurocholic acid (Fold change: 0.7756; AUC 0.8672), this phenomenon is consistent with Ping Li’s finding [22, 23].

β-Cryptoxanthin is converted to retinol (vitamin A), its health-promoting properties including antioxidant, antiobesity as well as the prevention of bone tissue loss [24]. In vitro studies showed that β-cryptoxanthin exhibited beneficial effects on bones, such as calcification and increased concentration of ALP, thus proving a potential anabolic effect on bone calcification [25]. The meta-analysis of observational studies published in 2021 found that high consumption of β-cryptoxanthin is associated with a lower risk of osteoporosis (OR = 0.79) and the occurrence of hip fractures (OR = 0.71) [26]. In our study, the patients with DDH had lower concentration of β-cryptoxanthin (Fold change: 0.4646; AUC 0.8784). This maybe the decreased level of β-cryptoxanthin induced the malfunction of bone metabolism.

Arabinosylhypoxanthine is part of organic compounds known as purine nucleosides. The final metabolite in the conversion of Arabinosyladenine-5’-monophosphate to arabinosyladenine then to Arabinosylhypoxanthine. Hypoxanthine is a purine derivative in reaction intermediate of adenosine metabolism. It is associated with an inborn error of metabolism (xanthinuria type 1). By combining the α-tocopherol and taurocholic acid, we could achieve the differential diagnosis of DDH (AUC = 0.935). These markers have the potential to become valuable diagnostic tools in the future.

## Conclusions

In this project, we used metabolomics to discover biomarkers of patients with DDH. The main differential metabolic pathways focused on arginine and proline metabolism, primary bile acid biosynthesis, phenylalanine metabolism and histidine metabolism. By combining the α-tocopherol and taurocholic acid, we could achieve the differential diagnosis of DDH (AUC = 0.935), these may become a diagnostic marker in the future. The specific pathology needs to be fully studied. This study has some shortcomings: (1) the small sample size from single center. (2) the further evaluation of these metabolites is needed. In summary, α-tocopherol and taurocholic acid are potential biomarkers of DDH.

### Electronic supplementary material

Below is the link to the electronic supplementary material.

Supplementary Material 1

## Data Availability

All data supporting the conclusions of this research article are included within the manuscript.

## Abbreviations

HC: Healthy control
UPLC: Ultraperformance liquid chromatography-QTOF: Quadrupole time-of-flight
MS: Mass spectrometry
PUMCH: Peking union medical college hospital
MRI: Magnetic resonance imaging
MTBE: Methyl tert-butyl ether
QC: Quality control
VIP: Variable importance in the projection
OPLS-DA: Orthogonal partial least-squares discriminant analysis
ROC: Receiver operating characteristic
SD: Standard deviation
FC: Fold Change
