# Supplementary material for: Plasma metabolomics signatures of developmental dysplasia of the hip in Tibet plateau
Source: Orphanet J Rare Dis. 2024 Jun 8;19:228. doi: 10.1186/s13023-024-03230-w (PMC11161931; doi:10.1186/s13023-024-03230-w)
Supplement: Supplementary file 1 — Supplementary Material 1 [file 13023_2024_3230_MOESM1_ESM.docx]

Reviewer #1: Authors addressed all these questions raised by reviewers.

**Author response:** Thanks for your comment.

Reviewer #2: Dear Author,

There is no novelty in your research to consider in context of already published scientific literature except just a demographic variation

**Author response:** Thanks for your comment.

Developmental dysplasia of the hip (DDH) is a common childhood health complaint, whose etiology is multifactorial. The incidence of DDH is variable and higher in Tibet plateau. Here, we collected plasma samples and studied the metabolomics signatures of DDH. This is a small scale exploration experiment, we will continue to verify these promising metabolites in the future.

Reviewer #3: the Discussion (line 302) : Could you make any match between clinical characteristics and metabolomics analisys? any impact?

**Author response:** We have added the information.

Ping Li et al found that taurocholic acid sodium hydrate could induce chondrogenesis and osteogenesis in mesenchymal stem cells (MSCs), ameliorating the secondary osteoporosis and delay the progression of intervertebral disc degeneration (IDD) in mice by targeting MAPK3 [22]. In our research, we also found that the patients with DDH had lower concentration of taurocholic acid (Fold change: 0.7756; AUC 0.8672), this phenomenon is consistent with Ping Li’s finding [22,23].

β-Cryptoxanthin is converted to retinol (vitamin A), its health-promoting properties including antioxidant, antiobesity as well as the prevention of [bone tissue](https://www.sciencedirect.com/topics/medicine-and-dentistry/bone-tissue) loss [24]. In vitro studies showed that β-cryptoxanthin exhibited beneficial effects on bones, such as calcification and increased concentration of ALP, thus proving a potential anabolic effect on bone calcification [25]. The meta-analysis of observational studies published in 2021 found that high consumption of β-cryptoxanthin is associated with a lower risk of osteoporosis (OR = 0.79) and the occurrence of hip fractures (OR = 0.71) [26]. In our study, the patients with DDH had lower concentration of β-cryptoxanthin (Fold change: 0.4646; AUC 0.8784). This maybe the decreased level of β-cryptoxanthin induced the malfunction of bone metabolism.

22. Li P, Chen Z, Meng K, Chen Y, Xu J, Xiang X, Wu X, Huang Z, Lai R, Li P, Lai Z, Ao X, Liu Z, Yang K, Bai X, Zhang Z. Discovery of Taurocholic Acid Sodium Hydrate as a Novel Repurposing Drug for Intervertebral Disc Degeneration by Targeting MAPK3. Orthop Surg. 2024;16(1):183-195.

23. Arai Y, Park H, Park S, Kim D, Baek I, Jeong L, Kim BJ, Park K, Lee D, Lee SH. Bile acid-based dual-functional prodrug nanoparticles for bone regeneration through hydrogen peroxide scavenging and osteogenic differentiation of mesenchymal stem cells. J Control Release. 2020; 328: 596-607.

24. Kulczyński B, Sidor A, Brzozowska A, Gramza-Michałowska A. The role of carotenoids in bone health-A narrative review. Nutrition. 2024; 119: 112306.

25. Michaëlsson K, Larsson SC. Circulating Alpha-Tocopherol Levels, Bone Mineral Density, and Fracture: Mendelian Randomization Study. Nutrients. 2021;13(6):1940.

26. Kim SJ, Anh NH, Diem NC, Park S, Cho YH, Long NP, Hwang IG, Lim J, Kwon SW. Effects of β-Cryptoxanthin on Improvement in Osteoporosis Risk: A Systematic Review and Meta-Analysis of Observational Studies. Foods. 2021;10(2):296.

**Changes and line numbers:** line number 330-349 of page number 20-21; line number 494-513 of page number 27-28.
